# The effects of pellet quality and pellet die thickness on growth performance of finishing pigs

**DOI:** 10.1093/tas/txag111

**Published:** 2026-07-22

**Authors:** Patrick A Badger, Haley K Otott, Charles R Stark, Adam Donnelly, Robert Goodband, Nelsa M Beckman, Allison K Blomme, Diego A Lopez, Chad B Paulk

**Affiliations:** Department of Grain Science and Industry, College of Agriculture, Kansas State University, Manhattan, KS 66506, United States; Department of Grain Science and Industry, College of Agriculture, Kansas State University, Manhattan, KS 66506, United States; Department of Grain Science and Industry, College of Agriculture, Kansas State University, Manhattan, KS 66506, United States; Borregaard USA, INC. Rothschild, WI 54474, United States; Department of Animal Sciences and Industry, Kansas State University, Manhattan, KS 66506, United States; Department of Grain Science and Industry, College of Agriculture, Kansas State University, Manhattan, KS 66506, United States; Department of Grain Science and Industry, College of Agriculture, Kansas State University, Manhattan, KS 66506, United States; Department of Grain Science and Industry, College of Agriculture, Kansas State University, Manhattan, KS 66506, United States; Department of Grain Science and Industry, College of Agriculture, Kansas State University, Manhattan, KS 66506, United States

**Keywords:** fines, finishing pigs, pellet die, pellet quality, pelleting

## Abstract

Two experiments were conducted to determine the effects of percentage pellet fines and pellet die thickness on finishing pig growth performance. In Exp. 1, 350 finishing pigs (600 × 241; DNA, Columbus, NE) were used over time across three separate weight ranges. In each of the three studies, there were 10 pigs per pen and seven pens per treatment. The three weight ranges were 44 to 68, 81 to 106, and 118 to 144 kg. Treatments consisted of a meal control diet and pelleted diets with 9 to 16, 41 to 47, 60 to 68, and 84 to 91% fines measured at the feeder. From 44 to 68 kg pigs, meal as percentage fines at the feeder increased, ADFI increased (linear, *P* = 0.006) and G: F decreased (linear, *P* = 0.002). Pigs fed the diet averaging 13% fines had increased (*P* < 0.05) G: F compared to those fed the meal diet. For 81 to 106 kg pigs, ADFI increased (linear, *P* = 0.016) and G: F decreased (linear, *P* < 0.001) as percentage fines at the feeder increased. Pigs fed pellets with an average of 13% fines had increased (*P* < 0.05) G: F compared to pigs fed the meal diet. From 118 to 144 kg pigs, ADFI increased (linear, *P* = 0.006) and G: F increased (linear, *P* = 0.002) as percentage fines at the feeder increased. Pigs fed pellets with up to 42% fines had improved (*P* < 0.05) G: F compared to pigs fed meal feed. In Exp. 2, 314 pigs (initially 52 kg) were used in a 76-day study to evaluate the effects of pellet die thickness on pig growth performance. Treatments consisted of a meal control diet or pelleted diets from a thick die effective length: diameter (L: D) ratio of 8.7 fed as-is, pellets from a thin die (L: D 5.5) fed as-is, screened thin die pellets (L: D 5.5), and screened pelleted fines fed as a meal. There were nine pigs per pen and seven pens per treatment. Overall, pigs fed thick die pellets (L: D 8.75, 28% fines) had increased (*P* < 0.05) ADG compared to those fed either meal, thin die pellets (L: D 5.5, 42% fines), or pellet fines. Pigs fed L: D 8.75 pellets had increased (*P* < 0.05) G: F compared to pigs fed the meal treatment. In conclusion, minimizing the amount of pellet fines present at the feeder increased G: F. Increasing pellet die thickness which reduced the amount of pellet fines at the feeder also led to an improvement in ADG and G: F compared to pigs fed meal diets, diets pelleted using the L: D 5.5, or pellet fines.

## Teaser

Pelleting swine diets improves feed efficiency in finishing pigs; however, this response is dependent on the amount of pellet fines at the feeder. Increasing the amount of pellet fines at the feeder results in poorer feed efficiency.

## Introduction

Diet costs account for the largest financial input for pork producers during the finishing phase of production. To mitigate the increased financial input for these phases of pig production, producers may elect to feed pelleted diets. Pelleting diets can improve bulk density of the diets, flowability, and pig growth performance. Feeding pigs pelleted diets increases G: F due to reduced feed wastage (Stark et al. 1994; Myers et al. 2013; Nemechek et al. 2015). Previous research has observed improvements of 5 and 8% for ADG and G: F, respectively, in pigs fed pelleted diets versus pigs fed meal diets (Paulk and Hancock 2015).

When pelleting swine diets, producing good quality pellets is important to optimize the growth performance response to pelleting. Stark et al. (1994) observed that as pellet fines measured at the feeder surpassed 30%, growth performance benefits associated with feeding pelleted diets decreased. Therefore, if pellets fines at the feeder increase, the benefits of pelleting may be reduced, negatively affecting G: F and overall profitability.

Improving pellet quality is a multifaceted approach. Pellet quality is influenced by conditioning temperature and time, steam characteristics, grain particle size, diet formulation, pellet die thickness, and pellet cooling conditions (Behnke 1994). Increasing pellet die thickness, increases the shear force applied on the feed as it is extruded through the pellet die and high compression increases binding capacity and improves pellet quality (Saensukjaroenphon 2019). However, this typically leads to a reduction in feed mill production rate. Therefore, the goal to pelleting swine diets is to find the optimal balance between achieving pellet quality and maintaining necessary production rates.

Increasing pellet die thickness increases the residence time of feed in the die which enhances pellet quality but also elevates the temperature of pellets as they exit the die (Saensukjaroenphon 2019). However, there has also been limited information on the impact of using various pellet die thicknesses or screening pellet fines on growth performance of pigs. Therefore, the objective of Exp. 1 was to determine if the response to pellet quality was dependent on the weight range of finishing pigs. Subsequently, our objective in Exp. 2 was to determine the influence of pellet die thickness and removing pellet fines on growth performance of finishing pigs. Our hypothesis was that reducing the amounts of fines present at the feeder would improve feed efficiency and that pellet die thickness would result in fewer fines at the feeder, therefore, also improving feed efficiency.

## Materials and methods

The experimental procedures followed in these experiments were approved by the Kansas State University Institutional Animal Care and Use Committee (IACUC # 4942).

### Experiment 1: 43 to 68, 81 to 106, and 118 to 144 kg pigs

A total of 350 growing pigs (Line 600 × 241; DNA, Columbus, NE, initially 43 kg) were used in a series of growth assays over the distinct weight ranges of 43 to 68, 81 to 106, and 118 to 144 kg lasting 20, 21, and 20 days, respectively. Pigs were housed with 10 pigs per pen and an equal number of barrows and gilts per pen. Pens measured 3.05 × 2.44 m, allowing 0.74 m^2^ per pig. Pens contained one cup waterer and one two-hole dry self-feeder to allow for ad libitum access to feed and water. Pigs were fed a common meal diet until the onset of the first experimental weight range. At the initiation of each experimental weight range, pens were weighed and randomly allotted to one of seven weight blocks. For each weight range, there were 35 pens and seven replications per treatment. Between each experimental weight range, pigs were fed a common diet for 10 days consisting of a pellet and meal blend. Treatment diets were delivered to each pen and recorded by the computerized feeding system (FeedPro; Feedlogic Corp., Willmar, MN). The feeding system had two hoppers and loaded one hopper with pellet fines and the other with pellets. At feed delivery, pellet fines and pellets were blended at the feeder. Treatments consisted of the meal control (not pelleted), screened pellets, 75% pellets and 25% fines, 50% pellets and 50% fines, and 25% pellets and 75% fines.

Pigs were fed a diet with the same nutrient composition, but with different diet forms (Table 1). The diet was formulated to meet or exceed NRC (NRC 2012) requirement estimates and manufactured in meal form (Hubbard Feeds; Beloit, KS). Corn was ground using a hammer mill (#7 screen, Champion Series Hammermill, Model 11.5 × 38, CPM/Roskamp Champion, Waterloo, IA) to achieve a target particle size of 500 to 600-µm. The diet was then delivered to the O.H. Kruse Feed Technology and Innovation Center (Manhattan, KS) for further processing. The feed was subdivided to create the meal control and the remaining feed was pelleted to create the pellet and fines treatments. Feed was steam conditioned (twin staff pre-conditioner, Model 150, Wenger, Sabetha, KS) to a target of 82°C and pelleted using a 30-horsepower pellet mill (1012-2 HD Master Model, California Pellet Mill, Crawfordsville, IN). The pellet mill die used was 4.8 × 31.8 mm (L: D 6.67) for the 43 to 68 and 81 to 106 kg weight ranges (Table 3). Pellet mill production rate was determined by collecting pellets for 30 s from the front of the mill and immediately weighed. For pigs in the 43 to 68 kg weight range, pelleted feed was conditioned at 82.7°C resulting in a hot pellet temperature of 89.1°C. Pellet mill production rate was approximately 918 kg/h. For the 81 to 106 kg weight range, pellets were steam conditioned at approximately 76.9°C which resulted in an approximate hot pellet temperature of 85.2°C. Pellet mill production rate was approximately 936 kg per h. For pigs in the 118 to 144 kg weight range, the pellet die thickness was increased to a 4.8 × 57.2 mm die (L: D 12) to match the approximate amount of fines for pelleted treatments at the feeder as the previous two weight ranges. This resulted in pelleted feed being steam conditioned at approximately 67.0°C and a hot pellet temperature of 81°C. Pellet mill production rate was decreased to approximately 702 kg per h. For all weight ranges, pellets were cooled (OP-FLO™ AIR COOLER; Bliss Industries, Ponca City, OK) and subsequently screened with a #5 (4.0 mm) screen (Gentle Roll; EBM Manufacturing, Inc. Norfolk, NE) to separate pellets and fines. Screened pellets were collected and predetermined amounts of pellets were then passed through a single pair crumble roll (Eco Roll 7; Cañon City, CO) twice to create fines that are similar in particle size to those created during pellet production to serve as the pellet fines that were blended to achieve the treatments diets.

**Table 1:** Composition of experimental diets, exp. 1 (as-fed basis).^1^

	Weight range, kg		
Item	43 to 68	81 to 106	118 to 144
**Ingredient, %**			
** Corn**	73.54	81.08	81.65
** Soybean meal (46.5% CP)**	21.23	14.15	13.8
** Corn oil**	1.50	1.50	1.50
** Calcium carbonate**	0.75	0.65	0.60
** Calcium phosphate (monocalcium P)**	0.50	0.28	0.10
** Sodium chloride**	0.50	0.55	0.50
** L-Lys-HCl**	0.45	0.39	0.38
** DL-Met**	0.11	0.06	0.09
** L-Thr**	0.14	0.12	0.16
** L-Trp**	0.04	0.04	0.04
** L-Val**	0.09	0.05	0.04
** Vitamin premix with phytase^2^**	0.25	0.25	0.25
** Trace mineral premix^3^**	0.15	0.15	0.15
** Pellet binder^4^**	0.75	0.75	0.75
**Total**	100	100	100
**Calculated analysis**			
**Standardized ileal digestible (SID) AA, %**			
** Lys**	1.05	0.83	0.81
** Ile: Lys**	55	55	56
** Leu: Lys**	123	135	138
** Met: Lys**	33	31	36
** Met and Cys: Lys**	55	56	62
** Thr: Lys**	60	61	68
** Trp: Lys**	19.2	19.1	19.3
** Val: Lys**	69	69	69
** His: Lys**	37	39	40
** ME, kcal/kg**	3,362	3,362	3,386
** NE, kcal/kg**	2,551	2,581	2,608
** CP, %**	16.9	14.3	13.9
** Ca, %**	0.54	0.54	0.38
** P, %**	0.45	0.44	0.33

1 Experimental diets were mixed at Hubbard Feeds (Beloit, KS) and shipped to O.H. Kruse Feed Technology and Innovation for pelleting. All treatments within experiments were fed the same formulation diet with respect to weight ranges (43 to 68, 81 to 106, 118 to 144 kg).

2 Provided per kilogram of vitamin premix: 165,346 IU Vitamin A, 661,376 IU Vitamin D_3_, 17,637 IU Vitamin E, 13 mg Vitamin B_12_, 1,323 mg menadione, 3,307 mg riboflavin, 11,023 mg d-Pantothenic Acid, 19,841 mg niacin, also contained Ronozyme HiPhos GT 20000, 1,226 FYT/kg with an estimated release of 0.14% standardized total tract P.

3 Provided per kilogram of trace mineral premix: of 73 g Fe from ferrous sulfate, 73 g Zn from zinc sulfate, 22 g Mn from manganous oxide, 11 g Cu from copper sulfate, 198 mg I from calcium iodate, 198 mg Se from sodium selenite.

4 Ameri-Bond 2X, Borregaard, Sarpsborg, Norway.

Pigs were weighed on days 0, 7, 14, and 20 or 21 to determine average daily gain (ADG), average daily feed intake (ADFI), and gain to feed ratio (G: F). The presence of pellet fines at the feeder were monitored for each weight range by probing three randomly selected feeders per treatment each week using a hollow tube probe. All feeder samples were collected by probing each feeder on the left side, center, and right side of the feeder hopper. Feed samples were then screened using a #5 screen (4.0 mm). The percentage fines were then calculated by dividing the amount of pellets remaining after screening by the total weight of the sample. Feed samples were analyzed for moisture (934.01), crude protein (990.03), crude fiber (978.10), ether extract (920.39 A), and ash (942.05) at a commercial laboratory (Ward Laboratories, Inc. Kearney, NE; Table 2).

**Table 2:** Chemical analysis of experimental diets (as-fed basis).^1^

			Percentage fines		
Treatment	Meal control^2^	Screened pellets^2^	25^2^	50^2^	75^2^
**43–68 kg**					
** Moisture**	11.48	12.17	11.66	11.91	11.47
** Dry matter**	88.52	87.83	88.34	88.09	88.53
** Crude protein**	16.4	16.1	16.7	16.9	16.5
** Crude fiber**	2.9	2.7	2.7	2.5	2.8
** Crude fat**	3.4	3.4	3.5	3.1	3.4
** Ash**	3.76	3.61	3.85	3.86	3.75
**81–106 kg**					
** Moisture**	10.68	11.01	11.26	11.5	11.7
** Dry matter**	89.32	88.99	88.74	88.5	88.3
** Crude protein**	13.4	12.7	12.5	13	12.7
** Crude fiber**	0.3	0.7	0.5	0.6	0.6
** Crude fat**	3.9	3.5	3.6	3.7	3.8
** Ash**	2.99	3.09	2.98	3.04	2.86
**116–144 kg**					
** Moisture**	12.79	12.86	13.45	12.77	12.96
** Dry matter**	87.21	87.14	86.55	87.23	87.04
** Crude protein**	13.7	13.2	13.1	13.2	13.3
** Crude fiber**	2.2	2.5	2.3	2.3	2.2
** Crude fat**	5.3	5.2	5.2	5	5
** Ash**	2.98	2.89	2.88	2.95	2.92

1 Treatment samples were collected at the feeder and analyzed by Ward Laboratories, INC in Kearney, NE.

2 Assigned treatment of either meal control, screened pellets, 25, 50, or 75% fines.

**Table 3:** Feed production records for pelleting experimental diets, exp. 1.^1^

	Weight range, kg		
Item	43 to 68^2^	81 to 106^2^	118 to 144^3^
**Production rate, kg/h**	918.0	936.0	702.0
**Conditioning temperature, °*C***	82.7	76.9	67.0
**Hot pellet temperature, °*C***	89.1	85.2	81.0

1 Pellets were manufactured by a 30-horsepower pellet mill (1012-2 HD Master Model, California Pellet Mill, Crawfordsville, IN).

2 43–68 and 81–106 kg diets were pelleted with a 4.8 × 31.8 mm die to (L: D 6.67).

3 118–144 kg diets were pelleted with 4.8 × 57.2 mm die (L: D 12).

### Experiment 2

A total of 314 pigs (600 × 241 DNA, Columbus, NE; initially 52.4 kg;) were used in a 76-day growth assay to determine the effects of pellet die thickness and removal of pellet fines on growth performance and carcass characteristics. A common diet was formulated to meet or exceed NRC (NRC 2012) requirement estimates for each of three dietary phases based on target pig weight with diet form as the only difference (Table 5). Treatment diets consisted of a meal control (not pelleted), diet pelleted using a 4.0 × 35 mm pellet mill die (L: D 8.75) fed as-is (no pellet screening), diets pelleted using a 4.0 × 22 mm pellet mill die (L: D 5.5) fed as-is, screened pellets from a 4.0 × 22 mm (L: D 5.5) pellet mill die, and pelleted fines that were made by using sifted fines and grinding pellets to be a similar particle sizes to the sifted fines. Corn was ground using a 3-high roller mill (9 × 24, RMS Roller-Grinder, Harrisburg, SD) to achieve a target particle size of 500 to 600-µm. Diets were then batched in a 907-kg dual ribbon mixer (Model MX-40, Hayes-Stolz Industrial Manufacturing Co., Burleson, TX) prior to either pelleting or sending to the research farm as meal feed. Pelleted treatments were steam conditioned for approximately 30 s (California Pellet Mill 18 INF 6.5) and subsequently pelleted using a 100-horsepower pellet mill (California Pellet Mill, 3016-4). Naturally created pellet fines were screened with a 3.89 mm screen (GentleRoll; EBM Manufacturing, Inc., Norfolk, NE) and mixed with mechanically created pellet fines from a 4.0 × 22 mm pellet mill die (L: D 5.5) that were ground to a similar particle size using the same 3-high roller mill used to grind the corn.

**Table 5:** Ingredient composition of experimental diets for exp. 2, as-fed basis.^1^

Ingredient, %	43–68 kg pigs		81–106 kg		116–144 kg
** Corn**	75.57		80.95		84.97
** Soybean meal, Dehull, Sol Extr**	20.69		15.83		12.03
** Soybean oil**	1.00		1.00		1.00
** Calcium carbonate**	0.75		0.75		0.60
** Calcium phosphate (monocalcium)**	0.45		0.30		0.10
** Sodium chloride**	0.50		0.50		0.50
** L-Lys-HCl**	0.40		0.35		0.35
** DL-Met**	0.11		0.07		0.07
** L-Thr**	0.14		0.12		0.12
** L-Trp**	0.03		0.03		0.03
** L-Val**	0.06		0.04		0.02
** Vitamin premix^2^**	0.15		0.13		0.10
** Trace mineral premix^3^**	0.15		0.13		0.10
** Phytase^4^**	0.02		0.02		0.02
**Total**	100		100		100
**Calculated analysis**					
**Standardized ileal digestible (SID) AA %**					
** Lys**	1.00		0.84		0.75
** Ile: Lys**	57		58		57
** Leu: Lys**	129		140		146
** Met: Lys**	34		34		35
** Met and Cys: Lys**	48		59		62
** Thr: Lys**	63		64		66
** Trp: Lys**	18.5		18.8		19.0
** Val: Lys**	69		70		69
** Hist: Lys**	49		41		41
**Total Lys, %**	1.12		0.94		0.84
**ME, kcal/kg**	3,366		3,379		3,393
**NE, kcal/kg**	2,513		2,552		2,584
**SID Lys: NE, g/Mcal**	3.92		3.25		2.87
**CP, %**	16.7		14.7		13.2
**Ca, %**	0.51		0.42		0.34
**P, %**	0.44		0.39		0.33
**Available P, %**	0.30		0.26		0.21

1 Experimental diets were batched and mixed using a 907 kg mixer (Model MX-40, Hayes-Stolz Industrial Manufacturing Co., Burleson, TX) at the O.H. Kruse Feed Manufacturing and Innovation Center. Diets were subsequently loaded out for delivery or pelleted with their respective dies (L: D 5.5 or 8.75).

2 Provided per kilogram of vitamin premix: 1,653,439 IU Vitamin A, 661,376 IU Vitamin D3, 17,637 IU Vitamin E, 13.2 mg Vitamin B12, 1,323 mg menadione, 3,307 mg riboflavin, 11,023 mg d-Pantothenic Acid, 19,841 mg niacin.

3 Provided per kilogram of trace mineral premix: of 73 g Fe from ferrous sulfate, 73 g Zn from zinc sulfate, 22 g Mn from manganous oxide, 11 g Cu from copper sulfate, 198 mg I from calcium iodate, 198 mg Se from sodium selenite.

4 Quantum Blue 10G, AB Vista, Plantation, FL with an estimated release of 0.14% standardized total track P.

Pelleted treatment diets were steam conditioned to approximately 83°C (Table 7). Pellet mill production rates were collected from the front of the pellet mill for 10 s and immediately weighed. Hot pellet temperatures were collected using a thermos with a thermometer placed in the lid with the highest temperature being recorded. Pellet mill electrical consumption was recorded at the time of pellet mill production rate and hot pellet temperature sample collection. During the pelleting run, 5 pellet samples were collected at evenly spaced times from the transition prior to the bucket elevator. Pellet durability index (PDI) was analyzed with the New Holmen 100 (TEKPRO, North Walsham, U.K.), 30 s test. Pellets were screened using a #6 (3.4 mm) sieve, 100-g of screened pellets were placed in the chamber and air agitated for 30 s. The machine was set to 70 mbar air pressure and outfitted with a tissue filter. Pellets were then removed from the chamber and sieved once more to remove any residual pellet fines and subsequently weighed. Pellet durability index scores were calculated by dividing the amount of pellets after testing and screening by the initial pellet sample weight. Pellet durability index samples were completed in duplicate.

As in Exp. 1, feed samples were collected weekly by probing three randomly selected feeders per treatment to analyze the percentage fines at the feeder. Sampling procedure was identical as described for Exp. 1. Samples were then mixed to create a representative composite sample for proximate analysis. Feed samples were analyzed for moisture (934.01), crude protein (990.03), crude fiber (978.10), NDF (Van Soest et al. 1991), ether extract (920.39 A), and ash (942.05) at a commercial laboratory (Ward Laboratories, Inc. Kearney, NE; Table 6).

**Table 6:** Chemical analysis of experimental diets (as-fed basis).^1^

			Diet Form		
Item	Meal	L: D 8.75^2^	L: D 5.5, as-is^3^	L: D 5.5, screened^3^^,^^4^	Fines^4^^,^^5^
**Phase 1**					
** Moisture, %**	10.17	10.20	10.34	10.36	9.81
** Dry matter, %**	89.83	89.80	89.66	89.64	90.19
** Crude protein, %**	15.8	16.3	15.7	16.5	15.3
** NDF, %^6^**	2.9	7.1	6.7	7.2	6.9
** Crude fat, %**	4.1	4.1	3.9	4.8	3.8
** Ash, %**	3.51	3.87	3.76	3.87	3.58
**Phase 2**					
** Moisture, %**	10.10	10.96	11.23	10.67	10.24
** Dry matter, %**	89.90	89.04	88.77	89.33	89.76
** Crude protein, %**	14.6	13.3	13.4	14.0	12.9
** NDF, %^6^**	9.5	7.3	8.8	6.8	7.1
** Crude fat, %**	4.7	4.7	4.6	5.2	4.2
** Ash, %**	3.26	2.94	3.09	3.16	3.02
**Phase 3**					
** Moisture, %**	10.24	10.43	11.20	10.84	11.32
** Dry matter, %**	89.76	89.57	88.80	89.16	88.68
** Crude protein, %**	12.9	12.2	12.3	12.7	12.1
** NDF, %^6^**	7.1	6.3	7.9	7.3	6.6
** Crude fat, %**	4.2	4.1	4.7	4.7	5.0
** Ash, %**	3.02	2.59	2.74	2.87	2.56

1 Treatment samples were analyzed by Ward Laboratories, INC in Kearney, NE.

2 Pelleted using a 4.0 × 35 mm (L: D 8.75) die.

3 Pellets extruded through a 4.0* × *22 mm die (L: D 5.5) and fed as is.

4 Pellets screened with a 3.89 mm Gentle Roll screen.

5 Pellets ground using a three high roller mill to create mechanically created pellet fines.

6 Neutral detergent fiber, %.

**Table 7:** Exp. 2 pellet mill production records.^1^

Diet form	L: D 8.75^2^	L: D 5.5, as is^3^	L: D 5.5, screened^3^^,^^4^
**Phase 1**			
** Production rate, MT/h^5^**	3.48	4.38	4.48
** Conditioning temperature, °*C***	82.5	82.1	82.3
** Hot pellet temperature, °*C***	84.0	82.8	82.8
** kWh**	13.3	11.0	10.6
** Pellet durability index, %^6^**	76.8	51.4	52.6
**Phase 2**			
** Production rate, MT/h^5^**	3.27	4.38	4.35
** Conditioning temperature, °*C***	82.5	82.3	82.3
** Hot pellet temperature, °*C***	83.8	82.7	82.4
** kWh**	13.9	10.2	10.3
** Pellet durability index, %^6^**	80.1	38.4	49.6
**Phase 3**			
** Production rate, MT/h^5^**	3.27	4.23	4.44
** Conditioning temperature, °*C***	82.6	81.7	82.8
** Hot pellet temperature, °*C***	85.2	82.0	82.8
** kWh**	14.1	10.7	10.0
** Pellet durability index, %^6^**	75.3	36.6	54.5

1 Pellets were steam conditioned (California Pellet Mill, 18 INF 6.5) and subsequently pelleted with a 100-horsepower pellet mill (California Pellet Mill, 3016-4).

2 Pelleted using a 4.0 × 35 mm (L: D 8.75) die.

3 Pelleted using a 4.0 × 22 mm (L: D 5.5).

4 Pellets sieved using a 3.98 mm screen.

5 Metric tonnes per hour.

6 Pellet durability index scores determined by a 30-s Holmen 100 test.

Pens of pigs were weighed on day 0 and divided into one of seven blocks based on average pen weight. Pen weights were collected approximately every 14 d. At the conclusion of the experiment (day 76), pens of pigs were weighed and then individual pig weights were collection for determining carcass yield. Pigs were transported approximately 3 h to a USDA inspected process facility (Triumph Foods; St. Joseph, MO) for carcass data collection. Carcass data consisted of hot carcass weight, loin depth (mm), backfat depth (mm), and percentage lean. Loin and backfat depths were measured with an optical probe (Fat-O-Meter, SFK, Herley, Denmark). Carcass yield was calculated by dividing individual pig hot carcass weight by the corresponding individual live pig weight collected at the farm. Percentage lean was calculated using a proprietary equation of the processing facility.

### Statistical analysis

For Exp. 1, data were analyzed as a randomized complete block design using the PROC-GLIMMIX procedure of SAS (SAS Institute, INC., Cary, NC). Pen was the experimental unit in all experiments with initial weight of pigs in the pen as the blocking factor. Dunnett’s test was performed with pigs fed meal diet serving as the control with pigs fed pelleted treatments compared to pigs fed the meal diet. Linear and quadratic contrasts were constructed with the actual percentage fines from feeders using the PROC ILM procedure for unequally spaced treatments. Because the meal diet was not manufactured using the pellet mill, it was not included in the linear and quadratic contrast. Each weight range was analyzed separately. Results were considered significant if *P* ≤ 0.05 and a tendency if *P ≤ *0.10.

For Exp. 2, data were analyzed as a randomized complete block design using the PROC-GLIMMIX procedure of SAS (SAS Institute, INC., Cary, NC). Pen was the experimental unit with weight block as the blocking factor for growth performance analysis. Carcass data was analyzed with individual pig measurements serving as the observational unit, pen as the experimental unit and average initial weight serving as the block. Pairwise comparison was used to analyze results from both the growth performances and carcass characteristics utilizing Tukey adjustment for carcass characteristic analysis. Results were considered significant if *P ≤ *0.05 and a tendency if *P ≤ *0.10.

## Results

### Experiment 1: 43 to 68 kg

The conditioning temperature was 83°C which resulted in a hot pellet temperature of 89°C (Table 3). Pellet mill production rate was 917 kg per h. Pelleted treatments resulted in 12.5, 46.4, 67.6, and 90.4% fines at the feeder for screened pellets, 25, 50, or 75% pellet fines treatments (Table 3). There was no evidence of difference in ADFI or ADG between pigs fed pelleted diets and those fed the meal diet. Pigs fed pellets containing increased percentage fines had increased (linear, *P* = 0.006) ADFI and decreased (linear, *P* = 0.0016) G: F. There were no changes observed in ADG. Pigs fed pellets with 12.5% fines had improved (*P* < 0.05) G: F compared to those fed the meal diet.

### Experiment 1: 81 to 106 kg

Diets were steam conditioned at 77°C, resulting in a hot pellet temperature of 85°C (Table 3). Pellet mill throughput was similar to Exp. 1: 43 to 68 kg at 936 kg per h.

Pelleted treatments resulted in 15.5, 43.6, 60.5, and 86.0% fines at the feeder for screened pellets, 25, 50, and 75% pellet fines treatments, respectively (Table 4). There was no evidence of a difference in ADG for pigs fed pelleted diets compared to those fed the meal diet. Pigs fed pellets with 86.0% fines showed tendency for increased (*P < *0.10) ADFI compared to those fed the meal diet. Pigs fed pelleted diets had an increased (linear, *P* = 0.016) ADFI as the percentage fines increased at the feeder. Pigs fed pellet diets with 15.5% fines had improved (*P* < 0.05) G: F when compared to those fed meal diets. Whereas pigs fed pellets containing 86.0% fines had a tendency for decreased (*P < *0.1) G: F compared to pigs fed the meal diet. Gain: feed ratio increased (linear, *P* = 0.001) as percentage fines at the feeder decreased.

**Table 4:** Effects of increasing pellet fines on growth performance of finishing pigs (exp. 1).^1^

			Percentage fines				Probability, *P* <^2^	
Item	Meal control^3^	Screened pellets^3^	25^3^	50^3^	75^3^	SEM	Linear	Quadratic
**43–68 kg**								
** Percentage fines^4^**	—-	12.5	46.4	67.6	90.4			
**BW, kg**								
** Day 0**	43.57	43.59	43.57	43.66	43.60	0.612	0.829	0.970
** Day 20**	67.75	67.71	68.22	67.71	68.16	0.776	0.627	0.895
**Days 0 to 20**								
** ADG, kg**	1.21	1.21	1.22	1.20	1.23	0.021	0.641	0.919
** ADFI, kg**	2.37	2.27	2.36	2.32	2.45	0.038	0.006	0.558
** G: F**	0.509	0.532*	0.520	0.519	0.502	0.006	0.002	0.544
**81–106 kg**								
** Percent fines** ^4^	—	15.5	43.6	60.5	86.0			
**BW, kg**								
** Day 0**	81.59	81.52	81.58	81.52	81.56	0.838	0.942	0.978
** Day 20**	105.36	106.46	105.47	105.76	106.06	1.125	0.682	0.279
**Days 0–21**								
** ADG, kg**	1.13	1.19	1.14	1.15	1.17	0.024	0.608	0.199
** ADFI, kg**	3.04	3.05	3.01	3.19	3.26^†^	0.072	0.016	0.388
** G: F**	0.372	0.390*	0.378	0.362	0.358^†^	0.004	<0.001	0.505
**116–144 kg**								
** Percent fines^2^**	—-	9.6	41.8	65.1	83.6			
**BW, kg**								
** Day 0**	118.45	118.44	118.40	118.36	118.36	1.292	0.876	0.975
** Day 20**	141.07	143.35	142.93	142.77	143.84^†^	1.542	0.802	0.370
**Days 0 to 21**								
** ADG, kg**	1.14	1.25	1.23	1.22	1.24	0.034	0.800	0.626
** ADFI, kg**	3.41	3.47	3.47	3.48	3.69^†^	0.083	0.12	0.182
** G: F**	0.333	0.359*	0.353*	0.350^†^	0.336	0.005	0.005	0.299

1 A 3 weight range experiment was conducted on finishing pigs, 43 to 68 kg lasting 20 days, 81 to 106 kg lasting 21 days, 118 to 144 kg lasting 20 days.

2 Dunnets test in SAS with contrasts created with fines percentages at the feeder for each experiment to compare pelleted treatments to meal diet.

3 Assigned treatment of either meal control, screened pellets, 25, 50, or 75% fines.

4 Feed samples were collected at the feeder and sifted using a #5 (4.0 mm) sieve. The weight (grams) were then divided by the total sample weight to determine the percentage of pellets and fines.

* Denotes a significant difference of *P ≤ *0.05 when treatments are compared to the meal control.

† Denotes a trend *P ≤ *0.10 when treatments are compared to the meal control.

### Experiment 1: 118 to 144 kg

Diets were pelleted on the same pellet mill as the previous two experiments; however, a pellet mill die change was needed to maintain consistent percentage of fines at the feeder as compared to the previous two experiments. A 4.8 × 57.2 mm (L: D 12) die was used to achieve a similar pellet quality as the previous two weight ranges (41 to 68 and 81 to 106 kg). During pelleting, feed was conditioned at 67°C, resulting in a hot pellet temperature of 81°C. The L: D 12 die resulted in a production rate of 702 kg/h (Table 3).

Pelleted treatments resulted in 9.6, 41.8, 65.1, and 83.6% fines at the feeder for screened pellets, 25, 50, and 75% fines treatments, respectively (Table 4). There was no evidence of a difference for ADG in pigs fed pelleted diets compared to those fed the meal diet. Pigs fed pellets with increasing percent fines had decreased (linear, *P = *0.01) G: F. Pigs fed pellets with 83.6% fines had a tendency for increased (*P < *0.1) ADFI compared to pigs fed the meal diet. Pelleted treatments containing 9.6 and 41.8% fines had improved (*P* < 0.05) G: F when compared to those fed the meal diet. Pigs fed 65.1% fines had a tendency for improved (*P < *0.10) G: F compared to pigs fed the meal diet.

### Experiment 2

All pelleted treatment diets were steam conditioned to approximately 82°C (Table 5). Diets pelleted using the L: D 5.5 die had a 31% increase in production rate and 24% reduction in kWh compared to those pelleted using the L: D 8.75 die. Diets pelleted using the L: D 5.5 resulted in a hot pellet temperature of 82.6°C, while those pelleted using the L: D 8.75 resulted in a hot pellet temperature of 84.3°C. However, diets pelleted using the L: D 5.5 die resulted in a 30% poorer PDI compared to those pelleted using the L: D 8.75 die.

From d 0 to 76, pelleted treatments yielded 28, 42, and 37% fines at the feeder for feed pelleted using pellet dies L: D 8.75, L: D 5.5 fed as-is, and L: D 5.5 screened, respectively (Table 8). Pigs fed L: D 8.75 pellets had increased (*P* < 0.05) d 76 BW compared pigs fed either meal or L: D 5.5 pellets fed as-is while pigs fed screened L: D 5.5 pellets and only pellet fines were intermediate. Overall, pigs fed L: D 8.75 pellets had increased (*P* < 0.05) ADG compared to pigs fed meal, L: D 5.5 pellets fed as-is, or pellet fines. There was no evidence of differences in ADFI among any of the treatments. Pigs fed L: D 8.75 pellets had increased (*P* < 0.05) G: F when compared to pigs fed meal or L: D 5.5 pellets as-is, while pigs fed screened L: D 5.5 pellets or fines were intermediate. Pigs fed L: D 8.75 pellets had increased (*P* < 0.05) HCW compared to pigs fed L: D 5.5 pellets as-is with pigs fed all other treatments intermediate. There was no evidence of differences for pigs fed any of the treatments for carcass yield, backfat thickness, loin depth, or percentage lean.

**Table 8:** The effects of pellet die thickness and pellet fines on growth performances of pigs from 52 to 146 kg.^1^

	Diet form						
	Meal	L: D 8.75^2^	L: D 5.5, as-is^3^	L: D 5.5, screened^3^^,^^4^	Fines^4^^,^^5^	SEM	Probability (*P<*)
**BW Day 76, kg**	140.23^c^	145.58^a^	141.00^b,c^	143.10^a,b^	143.39^a,b^	1.142	0.005
**Days 0 to 76**							
** Fines at feeder, %^6^**	–	27.82	42.10	36.90	–		
** ADG, kg**	1.15^c^	1.23^a^	1.16^c^	1.20^a,b^	1.18^b,c^	0.013	0.002
** ADFI, kg**	3.10	3.15	3.08	3.13	3.10	0.045	0.775
** G: F**	0.372^b^	0.389^a^	0.377^b^	0.383^a,b^	0.381^a,b^	0.004	0.041
**HCW,^7^ kg**	105.55^a,b^	108.24^a^	103.74^b^	106.79^a,b^	105.60^a,b^	1.140	0.052
**Carcass yield, %**	74.89	74.74	74.22	74.70	74.61	0.193	0.163
**Back fat thickness, mm**	17.39	17.47	16.70	17.20	16.92	0.394	0.613
**Loin depth, mm**	66.19	66.19	66.38	65.95	66.85	0.559	0.827
**Lean,^8^ %**	53.71	53.74	54.05	53.74	53.94	0.182	0.590

a,b,c Means within row with differing superscripts are significantly different (*P* < 0.05).

1 A total of 314 pigs (initially 52.39* ± *1.51 kg) were used in a 76-day growth assay to determine the effects of pellet die thickness on pig growth performance.

2 Pelleted using a 4.0 × 35 mm (L: D 8.75) die.

3 Pellets extruded through a 4.0* × *22 mm die (L: D 5.5) and fed as is.

4 Pellets screened with a 3.89 mm Gentle Roll screen.

5 Pellets ground using a three high roller mill to create mechanically created pellet fines.

6 Composite of 3 random feeder probes per treatment each week during feeding phase to determine presence of fines.

7 Hot carcass weight.

8 Percent lean calculated using a proprietary formula from the packing facility.

## Discussion

Pelleted treatments within Experiment 1 were targeted to have 0% (screened pellets), 25%, 50% and 75% fines; however, treatments resulted in 14% ± 3.8% more fines at the feeder than expected. This increase in fines at the feeder was relatively consistent across treatments, leading the authors to hypothesize that additional fines were generated during pellet handling. After pellets were screened, they were then discharged through a distributor and downspout into a loadout bin. From the loadout bin, pellets were transferred via a drag conveyor to the discharge chute where they were discharged into a feed truck and delivered to the Kansas State University Swine Teaching and Research Center, where they were unloaded via a boom auger into a feed bin. Pellets were subsequently transferred using an auger from the feed bin into the feed delivery system and then conveyed to the feeder. This multiple-stage handling and transfer of pellets likely contributed to mechanical degradation of pellets, resulting in an increased proportion of fines at the feeder compared to the target levels. Previous research has demonstrated that 12% fines can be created between the pellet cooler and load out and additional 10 to 12% fines can be generated when unloading a feed truck using a boom auger (De Jong et al. 2017).

Pelleting swine diets is commonly used to improve nutrient utilization, feed efficiency, feed handling characteristics and bulk density. Most of the improvement in feed efficiency due to pelleting can be attributed to a decrease in feed wastage (Stark et al. 1994). As a result, improvements in ADG and G: F have been observed for pigs fed pelleted diets compared to pigs fed meal diets ranging from 4 to 7% (Stark et al. 1994; Wondra et al. 1995; De Jong et al. 2016). However, improvements in ADG are more variable across studies. Myers et al. (2013) observed that providing pelleted diets resulted in no improvements in pig ADG, whereas Paulk and Hancock (2015) observed a 5% improvement in ADG for pigs fed pelleted diets compared to pigs fed meal diets.

In Exp. 1 reported herein, pigs were provided with pelleted diets with varying amounts of pellet fines. During the experiment, pigs that were fed pelleted diets with <16% fines had an increase in G: F in range of 4 to 9% compared to pigs fed meal diets. Previous studies have shown a range of improvements to G: F of 4% (De Jong et al. 2013) to 13% (Nemechek et al. 2015) in pigs fed pelleted diets with comparable amounts of pellet fines at the feeder to the present experiment.

Although previous research has demonstrated consistent improvements in feed efficiency when pigs are fed pelleted diets, the degree of this response depends on pellet quality or the percentage of fines at the feeder. Myers et al. (2013) observed that pigs presented pellets with > 30% fines at the feeder had decreased G: F compared to pigs fed meal diets. In contrast, recent research demonstrated an improvement in G: F when pigs were fed diets containing an average of 40% fines throughout the grow-finish period compared to those fed meal diets (Williams et al. 2021). Feeder design can also influence how pellet fines affect performance by impacting the ease with which pigs waste feed. The experiments conducted herein used a single two-hole dry self-feeder. However, the industry utilizes a variety of feeder designs (e.g., dry feeders, wet/dry feeders, round feeders) that may influence feed wastage.

Experimental diets from Exp. 1 and the published literature have historically created pellet quality treatments by blending screened pellets with created pellet fines. True pellet fines are small, broken pieces of pellets that can pass through a No. 6 or 5 screen for 4.0- and 4.8-mm diameter pellets, respectively. These fines result when pellets fracture during cooling, handling, transport, or feeding. The goal for Exp. 2 was to create treatments by pelleting diets using different die thicknesses or screening pellets prior to shipment to the farm. Therefore, two different pellet mill die thicknesses were used in the current study. In addition, when diets were pelleted using the thicker die in the last phase in Exp. 1, pigs had an 8% improvement in ADG compared to those fed mash diets. The authors chose to use two different die thicknesses to also determine if pellet die thickness could influence the response to ADG.

It was hypothesized that increasing die L: D ratio would reduce pellet fines at the feeder due to nearly doubling the retention time of feed spent in the die. This allows particles to adhere to each other and produce a better-quality pellet. This resulted in large numerical differences in pellet durability index scores and percentage fines at the feeder between the diets pelleted using the L: D 8.75 compared to the L: D 5.5. Throughout the experiment, diets pelleted using the L: D 8.75, L: D 5.5 as-is, or L: D 5.5 screened resulted in 27.8, 42.8, and 36.9% fines at the feeder. Pigs fed diets pelleted using the L: D 8.75 (27% fines) had improved G: F compared to those fed meal diets. This was similar to the results observed in Exp. 1. There was no evidence of difference in G: F between pigs fed diets pelleted using L: D 5.5 (42.8% fines) or screened pellets (36.9% fines), pellet fines and the meal control diet. This is similar to results of the 43 to 68 kg and 81 to 106 kg weight ranges in Exp. 1; however, for 118 to 144 kg, pigs fed up to 65% fines tended to have improved G: F compared to those fed meal diets.

Feed mills may use a mechanical sifter or screener to remove the fines from the pellets after cooling in order to provide a consistent product to customers. The screened fines are then returned to the pellet mill and can be up to 30% when the pellet mill is manufacturing poor quality pellets (California Pellet Mill Co., 2019). Although this may improve the final product, it reduces the production rate of the feed mill, due to having to re-pellet feed. In addition, previous research has reported that the recycling of fines into meal feed prior to pelleting may decrease pellet quality (Saensukjaroenphon et al. 2021). Therefore, keeping fines separated from pellets after pelleting and feeding them as-is may lead to improvements in manufacturing efficiencies. For the experiment conducted herein, screening pellets that were pelleted using the L: D 5.5 die after pellet cooling but prior to shipping feed to the farm still resulted in 36.9% fines at the feeder compared 42.8% fines at the feeder for pellets that were pelleted using the L: D 5.5 die and not screened. Pigs fed the screened pellets and those fed only pellet fines had numerical improvements in ADG and G: F compared to pigs fed the meal control and non-screened pellets. Therefore, keeping fines segregated from pellets and feeding separately could have practical economic implications for swine producers. However, further research is needed to determine the economic impact on the feed manufacturing process and to further define the response to feeding screened pellets and pellet fines separately. In Exp. 2 conducted herein, pigs fed diets pelleted using L: D 8.75 die had increased ADG and final BW compared to pigs fed meal or diets pelleted using the L: D 5.5 die fed as-is, respectively. The influence of pelleting diets on pig ADG has been variable among studies. Previous research has demonstrated similar improvements in gain when pigs are fed pelleted diets compared to the meal control (Nemechek et al. 2015, 2016; Paulk and Hancock 2015). However, there was no evidence of difference in ADG in pigs fed pellets in Exp. 1. Previous research also observed no differences in ADG when fed pelleted diets compared to a meal control (Myers et al. 2013; Ball et al. 2015; de Jong et al. 2016). In addition, Exp. 1 and previous research has demonstrated no evidence of differences in BW gain due to percentage fines at the feeder (Nemechek et al. 2015, 2016). Experiments 1 and 2 differed in their designs due to emphasis placed on specific weight range responses to pellet quality for Exp. 1, while Exp. 2 was designed to evaluate the effects of pellet die thicknesses across the entirety of the finishing phase. There were no differences in ADG across Exp. 1; however, when the L: D 12 pellet mill die was used in the heaviest weight range (116 to 144 kg), pigs fed pellets with 10% fines had a numerically greater ADG. However, pellet mill throughput with the L: D 12 pellet mill die was reduced by 25% compared to pellets produced with the L: D 6.67 die. The numerical difference in ADG served as a baseline of potential responses to increased pellet die thickness, which was tested in Exp. 2.

Pigs fed L: D 8.75 (27.82% fines) pellets had increased HCW by 3.8% compared to pigs fed L: D 5.5 pellets fed as-is (42.10% fines). Previous research has shown improvements in final BW and HCW for pigs fed pellet diets compared to pigs fed meal feed but no studies have shown differences in carcass characteristics between pelleted diets with varying levels of fines (Nemechek et al. 2015; Paulk and Hancock 2015; Williams et al. 2021). Overholt et al. (2016) observed a decrease in carcass yield for pigs fed pelleted diets and attributed the reduction in yield to the increase in fat thickness. However, no differences were observed for pigs fed pelleted treatments or meal for carcass yield, backfat thickness, loin depth, or percentage lean within the current experiment.

In conclusion, these experiments demonstrate reducing the amount of pellet fines at the feeder increases G: F in finishing pigs. Pigs weighing up to 106 kg had improved G: F when provided with minimal fines while pigs weighing up to 144 kg had improvements in G: F up to 65% fines showing this weight range of pigs are not as susceptible to poor pellet quality. These experiments support previous research with G: F responses while ADG responses were variable between experiments. Increasing pellet die thickness, and the subsequent reduction in pellet fines, increased pig G: F responses in both experiments.

## List of abbreviations

ADG: average daily gain
ADFI: average daily feed intake
G: F: gain: feed ratio
L: D: length: diameter ratio
